# Pyruvate dehydrogenase activity is required for optimal growth under lipid deficient conditions

**DOI:** 10.1186/2049-3002-2-S1-P58

**Published:** 2014-05-28

**Authors:** Kartik Rajagopalan, Ralph DeBerardinis

**Affiliations:** 1 Children’s Medical Center Research Institute, UT Southwestern, Dallas, TX, USA

## Background

Otto Warburg’s observation that tumor cells have increased rates of glucose uptake and lactate secretion in comparison to normal cells spawned his notion that tumors have dysfunctional mitochondria. However, in addition to metabolizing glucose to lactate, tumors *in vivo* exhibit mitochondrial glucose oxidation, indicating activity of pyruvate dehydrognase (PDH), which gates entry of glucose derived carbon into the tricarboxylic acid (TCA) cycle [1]. Here we establish a model wherein we suppress PDH activity using RNAi, thus abrogating the flux of glucose derived carbon into the TCA cycle and ultimately lipids, and determine the extent to which PDH activity and oxidative metabolism of glucose are required for the growth of cancer cells.

## Materials and methods

Small hairpin RNAs against the transcript encoding the catalytic subunit of PDH were cloned into a retroviral vector which allowed doxycycline-inducible control of expression. Metabolism of cancer cells was studied *in vitro* using a combination of stable isotope tracing and gas chromatography-mass spectrometry (GC-MS). 2D growth assays were performed in medium containing lipid-replete normal serum as well as serum in which lipids had been extracted using petroleum ether.

## Results

Cancer cells transferred the carbon tracer from glucose to TCA cycle metabolites as well as fatty acids. Suppression of PDH E1α protein levels resulted in a significant reduction of this transfer, indicating that these metabolic fluxes were dependent on PDH activity. Remarkably, abrogation of PDH activity limited growth exclusively under conditions in which cells were grown in media that contained delipidated serum. This growth deficit could be rescued if cells were provided with a mixture of the fatty acids palmitate and oleate.

## Conclusions

Cancer cells exhibit PDH activity that allows transfer of glucose carbon to citrate and the TCA cycle as well as ultimately into fatty acid synthesis. Importantly, suppression of PDH activity limits growth in conditions in which cancer cells do not have access to fatty acids. As availability of fatty acids may be compromised in rapidly proliferating tumors due to factors such as poor perfusion, it is plausible that PDH activity is required for optimal growth *in vivo*.
